# Modeling the spatiotemporal properties of crosstalk between RyR-mediated and IP_3_R-mediated local Ca^2+^ release

**DOI:** 10.3389/fcell.2026.1736729

**Published:** 2026-04-22

**Authors:** DeAnalisa C. Jones, Eric A. Sobie

**Affiliations:** Department of Pharmacological Sciences, Icahn School of Medicine at Mount Sinai, New York, NY, United States

**Keywords:** calcium signaling, calcium sparks, heart failure, IP3 receptor, mathematical modeling, ryanodine receptor, stochastic modeling

## Abstract

Ryanodine receptors (RyR) and IP_3_ receptors (IP_3_R) are Ca^2+^ release channels expressed on the endoplasmic/sarcoplasmic reticulum (ER/SR) membrane in various cell types. Both the spatial localization and the distinct gating properties of these channels contribute to the diverse cellular functions controlled by intracellular Ca^2+^ signaling. It is known that both RyR2s and IP_3_R2s are expressed on the SR membrane of ventricular cardiomyocytes and that the expression of IP_3_R2s on the SR is increased in cardiac diseases such as heart failure (HF), and evidence that Ca^2+^ release through IP_3_R2s can influence RyR2-mediated Ca^2+^ release in excitation-contraction coupling has been described. However, despite the suggested functional role for crosstalk between RyR2s and IP_3_R2s, especially under pathologic conditions, most previous mathematical models of cardiomyocyte Ca^2+^ signaling have accounted for only RyR2s in isolation. We hypothesized that the combined effects of (1) fragmentation and dispersion of RyR2s within calcium release units (CRUs) and (2) increased expression of IP_3_R2s that occur in HF promote pro-arrhythmic Ca^2+^ spark behavior, which may contribute to increased risk of arrhythmogenic Ca^2+^ wave formation and incidence of ventricular arrhythmias. We built a stochastic mathematical model of local SR Ca^2+^ release events—Ca^2+^ sparks—that incorporates both RyR2s and IP_3_R2s. This model considers the spatial arrangement of RyR2s and IP_3_R2s relative to one another based on published immunohistochemistry studies and the arrangement of RyR2s under HF and healthy control conditions based on super-resolution microscopy data. RyR2 and IP_3_R2 gating are modeled based on single channel patch clamp studies which show that (1) RyR2 gating is stochastic and depends on local cytosolic [Ca^2+^], JSR [Ca^2+^], and allosteric coupling, (2) IP_3_R2 gating is stochastic and depends primarily on local cytosolic [Ca^2+^] and [IP_3_], and the (3) RyR2 has a larger single channel Ca^2+^ current than the IP_3_R2. Our simulations show that Ca^2+^ spark probability increases with increasing IP_3_R2 expression in HF CRUs and IP_3_R2 expression mitigates differences in mean duration of and mean total Ca^2+^ released during Ca^2+^ sparks observed in simulations in which HF is modeled as fragmentation and dispersion of RyR2s within CRUs alone. Overall, this mathematical modeling study suggests that increased IP_3_R2 expression in the context of HF may contribute to pro-arrhythmic Ca^2+^ signaling via increased Ca^2+^ spark frequency but may also serve a compensatory function by countering changes in Ca^2+^ spark morphology that arise due to RyR2 remodeling within CRUs in HF.

## 1 Introduction

Type II ryanodine receptors (RyR2s) on the membrane of the sarcoplasmic reticulum (SR) are the primary Ca^2+^ channels associated with the intracellular Ca^2+^ release that leads to heart muscle contraction. Considerable experimental evidence, however, supports a role for crosstalk between RyR2s and the type II isoform of inositol 1,4,5-trisphosphate receptors (IP_3_R2s) in modulating excitation-contraction (EC) coupling in the heart, which may become especially important in the context of diseased cardiomyocytes such as those from hypertrophic or failing hearts. This mostly indirect experimental evidence can be summarized as follows: (1) Several studies have demonstrated that IP_3_R2s co-localize with RyR2s on the SR membrane (Lipp et al., 2000; Mackenzie et al., 2002; Harzheim et al., 2009; Wullschleger et al., 2017; Demydenko et al., 2021; Jin et al., 2022) and that IP_3_R2 expression is increased at SR junctions in heart failure (HF) (Go et al., 1995; Harzheim et al., 2009; Jin et al., 2022); (2) stimulating the IP_3_ pathway using various agents has positive ionotropic effects in cardiomyocytes (Lipp et al., 2000; Mackenzie et al., 2004; Zima and Blatter 2004; Li et al., 2005; Proven et al., 2006; Domeier et al., 2008; Harzheim et al., 2009; Demydenko et al., 2021), and (3) stimulating the IP_3_ pathway also increases diastolic Ca^2+^ spark frequency in cardiomyocytes (Lipp et al., 2000; Mackenzie et al., 2004; Zima and Blatter 2004; Li et al., 2005; Domeier et al., 2008; Harzheim et al., 2009; Horn et al., 2013; Wullschleger et al., 2017; Salvador and Egger 2018; Demydenko et al., 2021; Jin et al., 2022)—a phenomenon that can be pro-arrhythmogenic.

The goal of the work presented here is to build a stochastic mathematical model of local Ca^2+^ release—or Ca^2+^ sparks—that incorporates both RyR2s and IP_3_R2s to study the effects of RyR2-IP_3_R2 crosstalk on cardiac EC coupling and how this might change in HF. Our working hypothesis is that HF conditions may cause more frequent and/or sustained local Ca^2+^ release, which may increase a cell’s susceptibility to arrhythmogenic Ca^2+^ waves. We believe this may help us better understand on a subcellular level why an increased incidence of arrhythmogenic events is observed in patients with HF (Hallstrom et al., 1995; Shadman et al., 2015).

### 1.1 Subcellular changes that occur in HF

Several subcellular changes are hallmarks of failing cardiomyocytes. Activity of the sarco-/endoplasmic reticulum Ca^2+^-ATPase (SERCA) that pumps Ca^2+^ back into the SR after a Ca^2+^ spark is reduced in HF due to decreased SERCA expression (Hasenfuss 1998; Dash et al., 2001; Frisk et al., 2021; Jin et al., 2022) and enhanced inhibition of SERCA by phospholamban (PLB)—which binds to and limits SERCA’s activity (Schwinger et al., 1998; Dash et al., 2001). RyR2s become hyperactive or “leaky” in HF due to post-translational modifications like phosphorylation and oxidation (Dashwood et al., 2020; Dridi et al., 2020). Clusters of RyR2s within individual calcium release units (CRUs) become fragmented, forming smaller, more dispersed arrangements in hypertrophy and HF (Kolstad et al., 2018). Finally, IP_3_R2 expression is increased in HF as described below. Here, we model two changes that occur in HF that have not been considered together previously: (1) fragmentation and dispersion of RyR2s within CRUs and (2) increased IP_3_R2 expression at SR junctions.

#### 1.1.1 Remodeling of RyR2s within CRUs in HF

**FIGURE 1:**
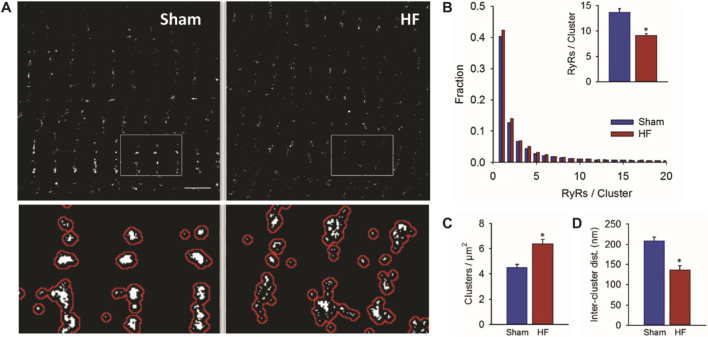
Super-resolution microscopy reveals fragmentation and dispersion of RyR2 clusters in failing rat ventricular myocytes. **(A)** RyR2 was fluorescently labelled in ventricular myocytes (VMs) from healthy, male Wistar-Hannover rats (Sham) and rats in which myocardial infarction and heart failure were induced using left coronary artery ligation (HF), and dSTORM imaging was performed. Compared to control (Sham) VMs, HF VMs had **(B)** fewer RyR2s/cluster, **(C)** more RyR2 clusters per µm^2^, and **(D)** shorter inter-cluster distances. Figure adapted from Kolstad et al. (2018).

Kolstad et al. imaged and quantified RyR2 clusters in healthy control (SHAM) and failing rat ventricular myocytes (HF) using direct stochastic optical reconstruction microscopy (dSTORM) (Kolstad et al., 2018). They found that fragmentation and dispersion of RyR2 clusters occurred in the HF group, resulting in fewer RyR2s per cluster, more RyR2 clusters per unit area, and shorter distances between clusters as demonstrated in Figure 1. Furthermore, they found that these changes contributed to dyssynchronous, pro-arrhythmic SR Ca^2+^ release in HF. These results are consistent with changes to RyR2 arrangements within CRUs in atrial fibrillation reported by Wullschleger et al. (2017) and increased incidence of uncoupled or “rogue” RyR2s in HF reported by others (Song et al., 2006; Dries et al., 2018; Jin et al., 2022).

#### 1.1.2 IP_3_R2 expression in HF

**FIGURE 2:**
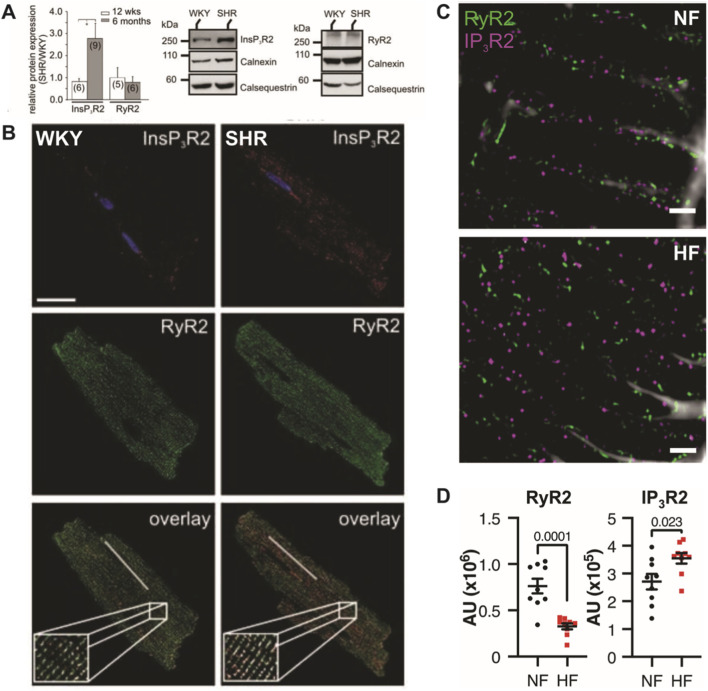
IP_3_R2s are expressed near RyR2s at SR junctions and this expression is increased in models of heart failure. Harzheim et al. isolated ventricular myocytes (VMs) from normotensive Wistar Kyoto rats (WKY) and from spontaneously hypertensive Wistar Kyoto rats (SHR) at 12 weeks and 6 months. **(A)** Protein levels were quantified using immunoblotting and showed increased relative IP_3_R2 protein levels in SHR compared to WKY at 6 months. No change was observed in RyR2 expression (left). Relative immunoblots at 6 months are shown for IP_3_R2 (middle) and RyR2 (right). **(B)** Immunofluorescent labeling of IP_3_R2 revealed higher IP_3_R2 expression near RyR2s on the SR in SHR compared to WKY VMs at 6 months. Panels **(A,B)** adapted from Harzheim et al. (2009). **(C)** Jin et al. fluorescently labelled RyR2 and IP_3_R2 in non-failing (NF) and failing (HF) VMs from human tissue samples, and stimulated emission depletion (STED) super-resolution microscopy was performed. **(D)** Immunoblotting showed that RyR2 protein expression is decreased in HF compared to NF VMs while IP_3_R2 expression is increased in HF compared to NF VMs. Panels **(C,D)** adapted from Jin et al. (2022).

Both RyR2s and IP_3_R2s are expressed on the SR of healthy cardiac myocytes; however, in ventricular myocytes, RyR2 expression is estimated to be 50-100x that of IP_3_R2 expression (Lipp et al., 2000; Zima and Blatter 2004; Horn et al., 2013; Hohendanner et al., 2014; Salvador and Egger 2018). Given this much greater expression of RyR2s, most SR Ca^2+^ release has been attributed to release via RyR2s. However, recent studies have shown that IP_3_R2 expression is increased in models of hypertrophy, ischemic dilated cardiomyopathy, and HF in human, rat, and mouse hearts (Mackenzie et al., 2002; 2004; Harzheim et al., 2009; Harzheim et al., 2010; Horn et al., 2013; Jin et al., 2022). Furthermore, several groups have used immunostaining to demonstrate that RyR2 and IP_3_R2 co-localize on the SR membrane of rat atrial and human, rat, and mouse ventricular myocytes (Lipp et al., 2000; Mackenzie et al., 2002; Mackenzie et al., 2004; Harzheim et al., 2009; Demydenko et al., 2021; Jin et al., 2022) and this co-localization is increased in hypertrophic (Harzheim et al., 2009) and failing (Go et al., 1995; Jin et al., 2022) ventricular myocytes (Figure 2).

### 1.2 Evidence for IP_3_R function in cardiac myocytes

Several studies have investigated a role for IP_3_R-mediated Ca^2+^ signaling in cardiac EC coupling. Stimulation of the IP_3_ pathway via endothelin-1 (ET-1) has been shown to have positive inotropic effects in atrial myocytes from mice and rats and ventricular myocytes from rats through larger Ca^2+^ transients and thus stronger contractions (Mackenzie et al., 2004; Li et al., 2005; Domeier et al., 2008; Harzheim et al., 2009; Horn et al., 2013). IP_3_R2 deficiency has been shown to be protective against pro-arrhythmic stress (Li et al., 2005; Horn et al., 2013; Hohendanner et al., 2014), and blocking IP_3_Rs with the antagonist 2-APB has been shown to prevent pro-arrhythmic Ca^2+^ signaling (Mackenzie et al., 2004). Stimulating IP_3_R2-mediated SR Ca^2+^ release with an IP_3_ ester has been shown to be pro-arrhythmic in atrial myocytes from rats (Lipp et al., 2000) and in hypertrophic rat ventricular myocytes and failing human left ventricular myocytes (Harzheim et al., 2009). Conversely, Blanch I Salvador and Egger showed that in mouse ventricular myocytes in which IP_3_R2 is overexpressed and RyR2 expression is decreased, spontaneous Ca^2+^ spark and wave occurrence is increased. Interestingly, however, when these cells are stimulated by endothelin-1, which activates IP_3_ release via the G_q_ pathway, Ca^2+^ wave occurrence is suppressed—a protective effect against arrhythmia (Salvador and Egger 2018). They attribute this to increased “eventless” Ca^2+^ leak via IP_3_R2s.

Diastolic SR Ca^2+^ sparks are typically infrequent in healthy ventricular myocytes, occurring approximately 50–100 times per cell per second (Cheng et al., 1993; Cheng and Lederer, 2008). This relatively low rate of spontaneous Ca^2+^ sparks, and the distances between RyR2 clusters generating the sparks, together minimize the likelihood that a Ca^2+^ spark will trigger cell-wide Ca^2+^ release (i.e., a potentially arrhythmogenic Ca^2+^ wave) outside of normal EC coupling. Several studies have shown, however, that these diastolic events become more frequent when the IP_3_ pathway is stimulated in healthy atrial myocytes, healthy ventricular myocytes, and hypertrophic ventricular myocytes (Lipp et al., 2000; Mackenzie et al., 2004; Zima and Blatter 2004; Domeier et al., 2008; Harzheim et al., 2010; Horn et al., 2013; Hohendanner et al., 2014). In hypertrophic ventricular myocytes in which IP_3_R2 expression is increased, diastolic sparks have higher amplitudes and faster rise times than events induced in wild-type (WT) myocytes (Harzheim et al., 2009).

“Eventless” IP_3_R-mediated SR Ca^2+^ leak—i.e. diastolic Ca^2+^ release not associated with observable sparks or puffs—has also been observed in healthy atrial myocytes and may have similar effects as “rogue” RyR2s which leak Ca^2+^ from the SR (Zima and Blatter 2004; Sobie et al., 2005; Song et al., 2006; Horn et al., 2013). This eventless IP_3_R2-mediated Ca^2+^ release leads to increased spark frequency, which may cause local [Ca^2+^] increases that either directly open RyR2s or promote RyR2 opening by increasing RyR2 sensitivity (Zima and Blatter 2004; Horn et al., 2013). Wullschleger et al. demonstrated that such crosstalk may be bidirectional (Wullschleger et al., 2017); when IP_3_R2 was overexpressed in mouse atrial myocytes, IP_3_-mediated SR Ca^2+^ release was able to induce RyR2-mediated SR Ca^2+^ release and *vice versa* (Wullschleger et al., 2017). These RyR2-mediated and IP_3_R2-mediated release events had the distinct spatiotemporal characteristics of “sparks” and “puffs,” respectively, and could be measured simultaneously (Wullschleger et al., 2017).

**FIGURE 3:**
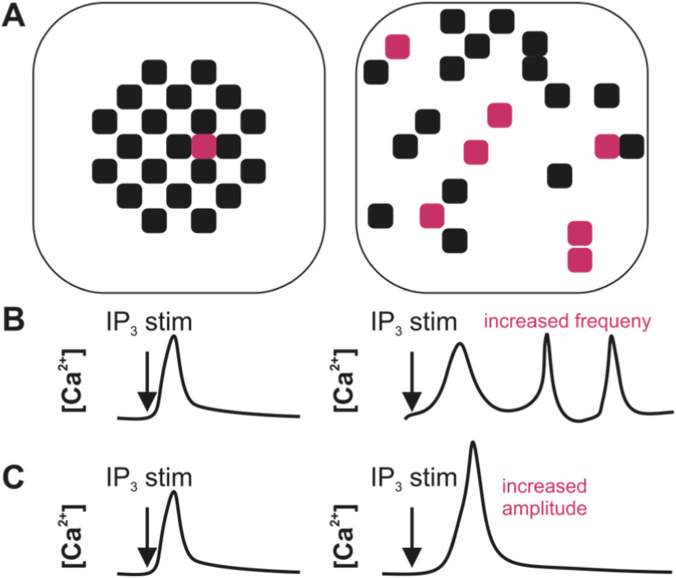
Model simulations in this study explored Ca^2+^ spark behavior in the presence of RyR2 fragmentation and dispersion and increased IP_3_R2 expression within CRUs, as seen in models of heart failure. We hypothesized that IP3 pathway stimulation under these conditions would result in more frequent, larger amplitude Ca^2+^ sparks. **(A)** Shown is an example of what an RyR2 arrangement within a HF CRU like compared to a healthy control CRU. In HF, RyR2s (black) become dispersed and IP_3_R2s (pink) become more highly expressed. **(B,C)** As suggested by several experimental studies, we expect changes to CRUs in HF to result in **(B)** increased spark frequency and **(C)** larger amplitude sparks in response to IP_3_ pathway stimulation. The specific number and arrangement of RyR2 and IP3R2 shown above are theoretical.

Based on the above experimental evidence, we hypothesize that remodeling of RyR2 arrangements and increased IP_3_R2 expression within CRUs in HF will result in more frequent, larger amplitude Ca^2+^ sparks upon IP_3_ pathway stimulation. This hypothesis is summarized by the schematics in Figure 3.

Despite indirect evidence for IP_3_R-mediated SR Ca^2+^ release in ventricular myocytes, neither puffs nor eventless IP_3_R-mediated Ca^2+^ release has been observed in these cells to date. This may be attributed to the low expression of IP_3_R2 in healthy ventricular myocytes compared to atrial myocytes (Lipp et al., 2000; Mackenzie et al., 2004; Zima and Blatter 2004; Domeier et al., 2008; Harzheim et al., 2010; Horn et al., 2013) as well as the low amplitude of puffs compared to sparks (Kockskämper et al., 2008; Wullschleger et al., 2017). If both RyR2-mediated and IP_3_R2-mediated SR Ca^2+^ release are occurring in the same cell, the low amplitude IP_3_R2-mediated events may be buffered or appear as noise in comparison to the higher amplitude RyR2-mediated events. However, across cardiac disease phenotypes, IP_3_R2 expression is increased in ventricular myocytes, and this may create a more prominent role for IP_3_R-mediated Ca^2+^ release events in these circumstances. Stated differently, it is possible that IP_3_R2s play a subtle role in normal ventricular myocyte function, which cannot be directly measured in experiments and thus warrants investigation through computational modeling. This role may become more pronounced in disease conditions.

To our knowledge, the first mathematical models to integrate both RyRs and IP_3_Rs into models of intracellular Ca^2+^ signaling in ventricular myocytes were two studies recently published from the same group (Hunt et al., 2020; Chung et al., 2023). The model published by Hunt et al. focused primarily on the role of IP_3_/IP_3_Rs on nuclear pro-hypertrophic signaling while the Chung et al. model and simulations are more relevant to our work. More specifically, this study presented a model of Ca^2+^ sparks which arise from both RyR2s and IP_3_R2s expressed within a ventricular myocyte dyad and showed that IP_3_R-mediated SR Ca^2+^ release increases spark probability by sensitizing RyR2s through increased baseline dyadic [Ca^2+^]. We seek to expand upon their findings here by modeling Ca^2+^ sparks via RyR2s and IP_3_R2s co-localized at SR junctions and investigating how changes observed in heart failure (HF)—such as RyR2 clustering remodeling and increased IP_3_R2 expression—impact Ca^2+^ spark behavior. Under such conditions, we found that IP_3_R2 expression within remodeled CRUs results in multi-spark events in which extra sparks occur in response to no trigger, indicating an increased propensity for diastolic Ca^2+^ spark activity in HF conditions.

## 2 Methods

### 2.1 RyR-IP_3_R Ca^2+^ spark model

**FIGURE 4:**
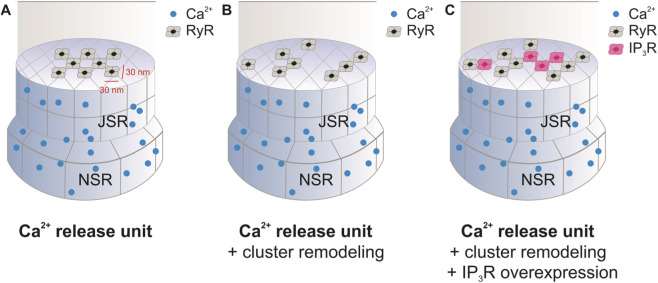
Changes observed in heart failure were incorporated into our **(A)** spatial Ca^2+^ release unit (CRU) model in two steps: **(B)** RyR2 cluster fragmentation and dispersion were first incorporated into this model and then **(C)** IP_3_R2s were interspersed with increasing expression amongst the RyR2s.

To investigate RyR2-IP_3_R2 crosstalk in the context of disease, we used the stochastic mathematical model developed by our group previously (Jones 2023) in which the positions of individual RyR2s within a Ca^2+^ release unit (CRU) can be manipulated within an (x,y) plane (Figure 4A) and then Ca^2+^ sparks can be simulated from that specific arrangement, similar to work by Cannell et al., Walker et al., and others (Cannell et al., 2013; Walker et al., 2014; Cosi et al., 2019; Iaparov et al., 2021; Mesa et al., 2022). To set the spatial arrangements of RyR2s within CRUs observed in CRTL and HF conditions, we relied on dSTORM images of RyR2s obtained from ventricular myocytes from healthy and spontaneously hypertensive rats (SHR) expressing a heart failure phenotype (Kolstad et al., 2018) (Figure 4B). Processing of these images and determination of individual RyR2 positions are described in detail elsewhere (Jones 2023). We then incorporated IP_3_R2s into the model (Figure 4C). Given that data on specific locations of individual IP_3_R2s relative to RyR2s are limited—i.e. we only know that they are expressed at SR junctions near RyR2s and that this expression is increased in HF—IP_3_R2s were positioned at random (x,y) locations within CRUs in each simulation using the *randi* function in MATLAB.

Changes in dyadic subspace and JSR [Ca^2+^] can be described as 2D reaction diffusion processes of which we solve discrete approximations (Equations 1,2 [Disp-formula e2]). *J*_*RyR*_ *(x,y)* and *J*_*IP3R*_ *(x,y)* represent the flux of Ca^2+^ from the JSR to the dyad at spatial location (x,y) via RyR2s and IP_3_R2s, respectively (Equations 3,4 [Disp-formula e4]). *n*_*open,RyR*_ *(x,y)* and *n*_*open,IP3R*_ *(x,y)* in Equations 3,4, [Disp-formula e4] refer, respectively, to the number of open RyR2s or IP_3_R2s at (x,y) at time *t*. P_RyR_ and P_IP3R_ are the respective single channel permeability constants to Ca^2+^ assuming a single RyR2 channel current of 0.16 pA and single channel IP_3_R2 current of 0.05 pA (see below), and *V*_*ds*_ *(x,y)* is the dyadic space sub-volume element at location (x,y), which is set to 1.8 × 10^−14^ μL for all simulations. *J*_*buffer,*i_ represents the buffering of Ca^2+^ in the dyadic space by calmodulin, the sarcolemmal membrane, or the SR membrane (Equation 5). *J*_*buffer, total*_ is the sum of all these buffering systems (Equation 6). *J*_*refill*_ *(x,y)* describes the refill of [Ca^2+^]_JSR_ from a constant, bulk NSR store of 850 μM with a time constant (τ_refill_) of 8 m (Equation 7) (Sobie et al., 2005). RyR2 gating, IP_3_R2 gating, and other considerations for modeling IP_3_R2 behavior are discussed below. Values used for each variable in Equations 1–7 are listed in Supplementary Table S1 and published in our previous work (Jones 2023).

$$\frac{\partial {\left[{Ca}^{2+}\right]}_{ds}\left(x,y\right)}{\partial t}=D_{myo}\frac{\partial^{2}{\left[{Ca}^{2+}\right]}_{ds}\left(x,y\right)}{{\partial x}^{2}}+\frac{\partial^{2}{\left[{Ca}^{2+}\right]}_{ds}\left(x,y\right)}{{\partial y}^{2}}+J_{RyR}\left(x,y\right)+J_{IP3R}\left(x,y\right)+J_{buffer,total}\left(x,y\right)$$ (1)

$$\frac{\partial {\left[{Ca}^{2+}\right]}_{JSR}\left(x,y\right)}{\partial t}=\beta \left[J_{refill}\left(x,y\right)+D_{JSR}\frac{\partial^{2}{\left[{Ca}^{2+}\right]}_{JSR}\left(x,y\right)}{{\partial x}^{2}}+\frac{\partial^{2}{\left[{Ca}^{2+}\right]}_{JSR}\left(x,y\right)}{{\partial y}^{2}}-J_{RyR}\left(x,y\right)-J_{IP3R}\left(x,y\right)\right]$$ (2)

$$J_{RyR}\left(x,y\right)=n_{open}\left(x,y\right)\cdot P_{RyR}\cdot \left[\frac{C_{JSR}\left(x,y\right)-C_{ds}\left(x,y\right)}{V_{ds}\left(x,y\right)}\right]$$ (3)

$$J_{IP3R}\left(x,y\right)=n_{open}\left(x,y\right)\cdot P_{IP3R}\cdot \left[\frac{C_{JSR}\left(x,y\right)-C_{ds}\left(x,y\right)}{V_{ds}\left(x,y\right)}\right]$$ (4)

$$J_{buffer,i}=k_{on,i}\cdot B_{i}\left(x,y\right)\cdot C_{ds}\left(x,y\right)-k_{off,i}\left[B_{total,i}-B_{i}\left(x,y\right)\right]$$ (5)

$$J_{buffer,total}=\sum_{i=1}^{i=3}J_{buffer,i}$$ (6)

$$J_{refill}\left(x,y\right)=\frac{{\left[{Ca}^{2+}\right]}_{NSR}-{\left[{Ca}^{2+}\right]}_{JSR}\left(x,y\right)}{\tau_{refill}}$$ (7)

### 2.2 RyR2 gating

A two-state, stochastic RyR2 gating model (Figure 6A) is used in this model where the transitions between the open and closed state of the RyR2 at location (x,y) is determined by Equations 8–12. After the opening of one RyR2 channel at time *t*, the probability of each individual RyR2 channel to open or close at the following time step *t = t + dt* is determined by *p*_*increase*_ *(x,y)* for closed channels and *p*_*decrease*_ *(x,y)* for open channels. *k* ^*+*^_*r*_ *(x,y)* and *k* ^*-*^_*r*_ *(x,y)* are the opening and closing transition rate constants. We also implement allosteric coupling between RyR2s through the term *k*_*coup*_^*num_net(x,y)*^. Although the functional significance of coupling remains largely unclear, compelling evidence supports its existence, as discussed in a recent comprehensive review (Rios, 2026). The coupling term *k*_*coup*_^*num_net(x,y)*^ is calculated as the exponential of the coupling constant, *EJ* (Sobie et al., 2005). For every channel, located at location (x,y), *num_net(x,y)* is calculated by determining the number of neighboring channels one has and how many of those neighbors are open versus closed, as described in further detail previously (Sobie et al., 2005; Ramay et al., 2011; Jones 2023). Constant variables for RyR2 gating are published in Jones (2023).

$$K_{r}\left(x,y\right)=K_{r,\max}-\alpha_{r}\cdot C_{JSR}\left(x,y\right)$$ (8)

$$k^{+}{\;}_{r}\left(x,y\right)=\frac{k^{+}{\;}_{r,\max}\cdot {C_{ds}\left(x,y\right)}^{hill}}{{C_{ds}\left(x,y\right)}^{hill}+{K_{r}\left(x,y\right)}^{hill}}$$ (9)

$$k_{coup}=e^{EJ}$$ (10)

$$p_{increase}\left(x,y\right)=dt\cdot n_{closed}\left(x,y\right)\cdot k^{+}{\;}_{r}\left(x,y\right)\cdot {k_{coup}}^{num\_net\left(x,y\right)}$$ (11)

$$p_{decrease}\left(x,y\right)=dt\cdot n_{open}\left(x,y\right)\cdot k^{-}{\;}_{r}\left(x,y\right)\cdot {k_{coup}}^{num\_net\left(x,y\right)}$$ (12)

### 2.3 Single channel current

While lipid bilayer experiments have given us confidence in the range of single channel RyR2 current (Mejía-Alvarez et al., 1999; Kettlun et al., 2003; Guo et al., 2012), similar data for IP_3_R2s are not available. The consensus seems to be that IP_3_Rs carry less current than RyRs, but precise current measurements have not been made. Smith and Parker estimate a single channel current of about 0.05 pA from “blip” (Ca^2+^ release event from one IP_3_R opening) recordings while Demydenko et al. states that RyR conductance is approximately 3x that of IP_3_Rs (Demydenko et al., 2021). In our simulations, we use a single channel RyR2 current of 0.16 pA (assuming diastolic SR [Ca^2+^] of 850 µM) and a single channel IP_3_R2 current of 0.05 pA.

### 2.4 IP_3_R2 gating

**FIGURE 5:**
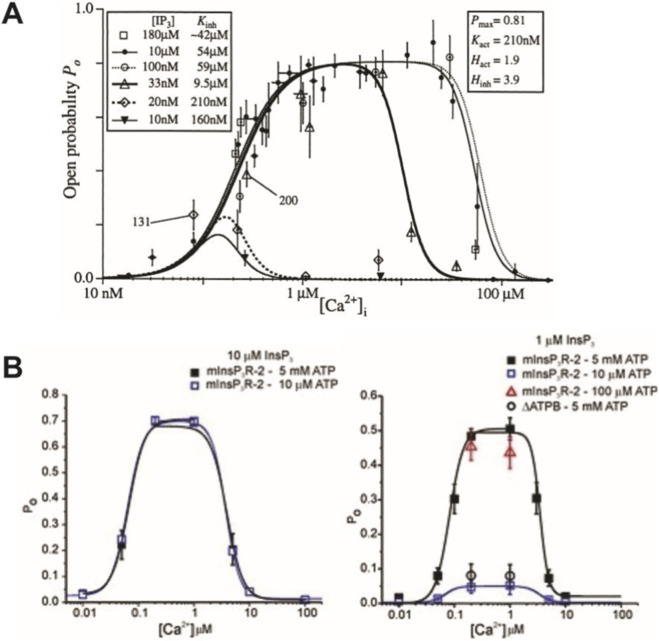
Patch clamp studies demonstrate that IP_3_R open probability (P_O_) increases with increasing [IP_3_] and is highest at intermediate intracellular [Ca^2+^]. **(A)** Mak et al. showed that nuclear IP_3_R1 P_O_ increases with increasing [IP_3_] up to 0.33 μM and peaks at intermediate intracellular [Ca^2+^] in *Xenopus* oocytes. Maximum P_O_ was found to be 0.81. Panel adapted from (Mak et al., 1998). **(B)** Wagner et al. showed a similar trend in IP_3_R2-expressing DT40 nuclei in which maximum IP_3_R P_O_ was around 0.5 for [IP_3_] = 1 μM and closer to 0.7 for [IP_3_] = 10 μM. In both cases, P_O_ was highest when intracellular [Ca^2+^] was between 0.1 and 10 μM. Panel adapted from (Wagner and Yule 2012).

IP_3_R2 gating differs from RyR2 gating in several ways: (1) IP_3_R2 requires the presence of IP_3_ to open and conduct Ca^2+^; (2) IP_3_R2 open probability increases with increasing [IP_3_]; (3) IP_3_R2 open probability is low at low intracellular [Ca^2+^], peaks at intermediate [Ca^2+^], and becomes low again at high [Ca^2+^]; (4) Opening and closing of IP_3_R2s is not known to exhibit allosteric coupling. IP_3_ and Ca^2+^-dependent behavior of IP_3_R2s is demonstrated by patch clamp studies in Figure 5.

**FIGURE 6:**
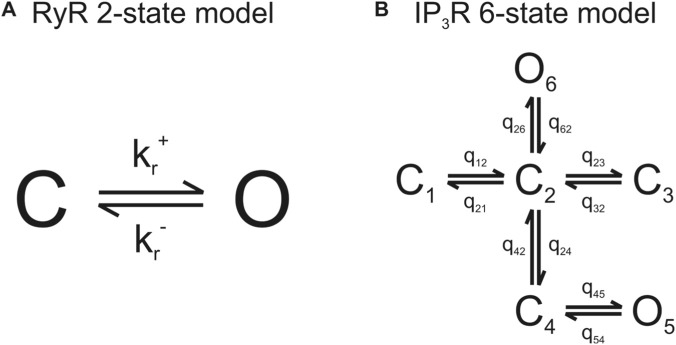
Markov models of RyR2 and IP_3_R2 gating were used in this study. **(A)** A two-state model in which RyR2 can be in the closed or open state is used in this RyR-IP_3_R crosstalk model (Sobie et al., 2002; Ramay et al., 2011; Song et al., 2016). **(B)** For the IP_3_R2 gating, a six-state model of type I and II IP_3_Rs published by Siekmann et al. (Siekmann et al., 2012) with six possible open and closed states was used.

A six-state Markov model developed by Siekmann et al. based on single IP_3_R2 experimental data (Siekmann et al., 2012; Cao et al., 2013) was incorporated into our model (Figure 6B).

In this model, an IP_3_R can exist in six states: two open and four closed. These states are further subdivided into two modes: park mode and drive mode. In park mode, the channel has a low open probability while it has a higher open probability in drive mode (Siekmann et al., 2012). Transitions between park and drive mode are described by q_24_ and q_42_ (Equations 13, 14), where m_24_, h_24_, m_42_, and h_42_ are intracellular [Ca^2+^] and [IP_3_]-dependent and a_24_, V_24_, and a_42_ are [IP_3_]-dependent only (Cao et al., 2013). V_42_ and other transition rates are constant and their values are listed in Table 1.

$$q_{24}=a_{24}+V_{24}\;\left(1-m_{24}h_{24}\right)$$ (13)

$$q_{42}=a_{42}+V_{24}m_{24}h_{24}$$ (14)

**TABLE 1:** Experimental findings indirectly support the notion of RyR-IP_3_R crosstalk in cardiomyocytes.

Study	Cell type (s)	IP_3_R2 and RyR2 co-localize on the SR	IP_3_R2 expression increased in disease	Stimulating IP_3_ pathway increases spark frequency	Stimulating IP_3_ pathway has positive ionotropic effects	IP_3_R2-mediated can cause RyR2-mediated release	IP_3_R2-deficiency protects against pro-arrhythmic events
Go et al. (1995)	Human VM	​	X	​	​	​	​
Lipp et al. (2000)	Rat AM	X	​	X	X	​	​
Mackenzie et al. (2002)	Rat AM	X	​	​	​	​	​
Mackenzie et al. (2004)	Rat AM Rat VM	​	​	X	X	​	​
Zima and Blatter (2004)	Cat AM Cat VM	​	​	X	X	​	​
Li et al. (2005)	Mouse AM	​	​	X	X	​	X
Proven et al. (2006)	Rat VM	​	​	​	X	​	​
Domeier et al. (2008)	Rabbit VM	​	​	X	X	​	​
Harzheim et al. (2009)	Mouse VM Rat VM Human VM	X	X	X	X	​	​
Harzheim et al. (2010)	Rat VM Human VM	​	​	​	​	​	​
Horn et al. (2013)	Mouse AM	​	​	X	​	​	​
Wullschleger et al. (2017)	Mouse AM	X	​	X	​	X	​
Blanch i Salvador and Egger 2018	Mouse VM	​	​	X	​	​	​
Demydenko et al. (2021)	Rat VM	X	​	X	X	​	​
Jin et al. (2022)	Human VM	X	X	X	​	​	​

Key experimental evidence to support RyR-IP_3_R, crosstalk in cardiomyocytes includes IP_3_R2 and RyR2 co-localize on the SR, increased IP_3_R2 expression in diseased cardiomyocytes, increased spark frequency and positive ionotropic effects in response to IP_3_ pathway stimulation using various stimuli, IP_3_R-mediated release causing RyR-mediated release and *vice versa*, and IP_3_R2 deficiency protecting against pro-arrhythmogenic Ca^2+^ release events. In the row corresponding to each published study, findings to support IP_3_R-RyR crosstalk are denoted with an “X” and the species in which these were found is listed.

**FIGURE 7:**
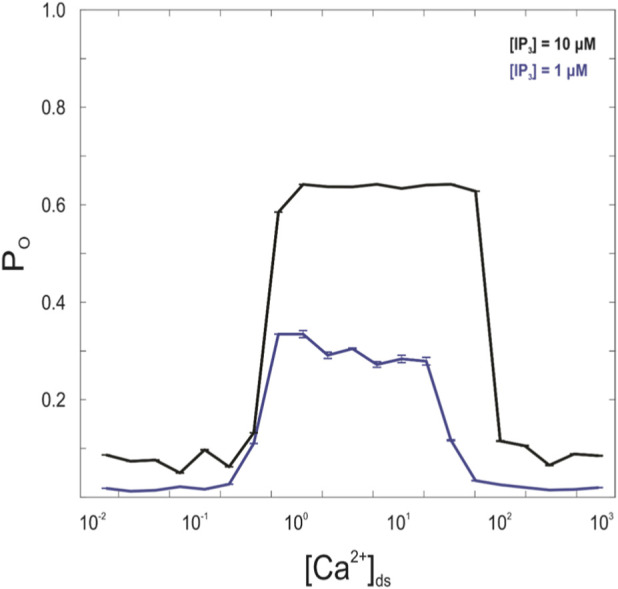
In the six-state IP_3_R gating model used in this study, peak opening probability (P_O_) occurred at high [IP_3_] and intermediate dyadic space [Ca^2+^]. Peak P_O_ for [IP_3_] = 1 μM occurs between [Ca^2+^]_ds_ = 1 μM and 10 μM. Peak P_O_ for [IP_3_] = 10 μM occurs between [Ca^2+^]_ds_ = 1 μM and 100 μM.

The open probability of IP_3_R2 was assessed by varying the concentrations of [IP_3_] and [Ca^2+^] while keeping N_IP3R_ at 1 and setting N_RyR_ and J_IP3R_ to 0 to avoid changes in intracellular [Ca^2+^] (Figure 7). Intracellular [Ca^2+^] was incrementally increased from 0.01 μM to 1,000 μM for both [IP_3_] = 1 μM and 10 μM. The IP_3_R2 was allowed to transition between states for 1,000 m according to the model gating, and the percentage of time the channel was in an open state was recorded as the open probability (P_O_). P_O_ was highest at high intracellular [IP_3_] and intermediate intracellular [Ca^2+^] consistent with data observed by Siekmann et al., 2012.

### 2.5 Characterizing Ca^2+^ spark behavior

For each simulation, one RyR2 within a Ca^2+^ release unit (CRU) was opened. If this opening triggered at least 4 other RyR2s and/or IP_3_R2s to open (Σ *n*_*open,RyR*_ *+* Σ *n*_*open,IP3R*_ > 5) within the simulation time, the event was considered a “spark.” The fraction of simulations that result in a spark out of all simulations run under a given set of conditions is reported as the spark fidelity. Resulting sparks were then characterized by (1) the total amount of Ca^2+^ released during the simulation time (Ca^2+^ mass in femtocoulombs, fC), calculated by taking the area under the curve of total current versus time; (2) the duration of the spark (in milliseconds, ms), defined by the time delay between when [Ca^2+^]_dyad_ exceeds and falls below 10% of its maximum; (3) and the maximum number of channels simultaneously open during the spark.

## 3 Results

### 3.1 Ca^2+^ spark fidelity increases with increasing N_IP3R_ and [IP_3_]

**FIGURE 8:**
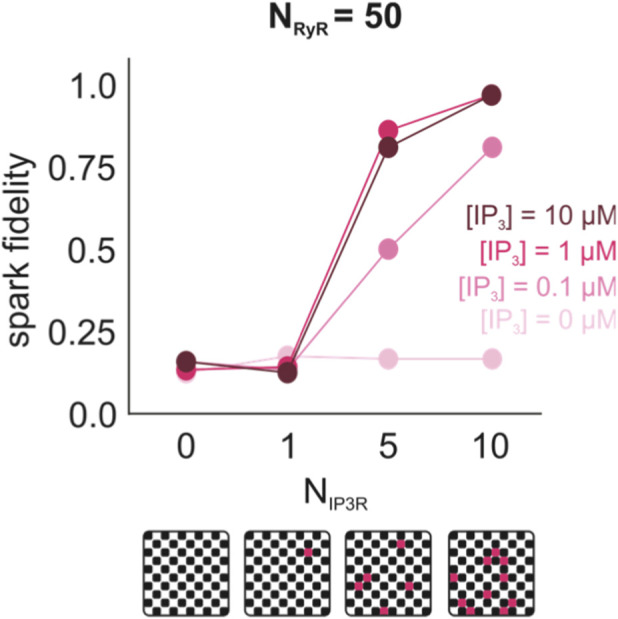
Spark fidelity increases with increasing N_IP3R_ and [IP_3_]. N_IP3R_ was varied from 0 to 10 and [IP_3_] was varied from 0 to 10 µM. For each set of conditions, 100 simulations were run and spark fidelity was measured (n_open_ >5 in response to opening one RyR2). Changes in spark fidelity are only observed when NIP3R > 0 and [IP3] > 0.

To assess the effect of IP_3_R2 expression near RyR2s at SR junctions, an initial set of simulations was run in which the number of IP_3_R2s (N_IP3R_) in a Ca^2+^ release unit (CRU) was increased from 0 to 10 while the number of RyR2s (N_RyR_) was kept constant at 50. For simplicity, in this set of simulations, RyR2s were arranged in a checkerboard similarly to what is often observed in healthy ventricular myocytes (Cabra et al., 2016; Kolstad et al., 2018), and IP_3_R2s were interspersed among the RyR2s at random locations (Figure 8, *bottom panel*). For each N_IP3R_ and [IP_3_], spark fidelity—the number of times opening one RyR2 resulted in a spark out of 100 trials—was recorded (Figure 8, *top panel*). These simulations showed that spark fidelity increases with increasing N_IP3R_ and that this trend is exaggerated at higher [IP_3_]. These results are consistent with experimental findings that stimulating the IP_3_ pathway increases spark frequency (see Table 2) and a recent modeling study by Chung et al. which showed that increasing N_IP3R_ increases spark probability (Chung et al., 2023).

**TABLE 2:** Six-state IP_3_R2 model fixed transition rates.

Parameter	Values (ms^-1^)
V_42_	100
q_12_	1.14
q_21_	0.0958
q_23_	4.75 × 10^−3^
q_26_	10.1
q_32_	0.0119
q_45_	4.14 × 10^−3^
q_54_	3.42
q_62_	3.27

Constant variables describing the six-state model transitions are listed. Further explanations can be found in the Supplementary Material for Siekmann et al. (2012), Cao et al. (2013).

### 3.2 IP_3_R2 expression within CRUs alters Ca^2+^ sparks in heart failure

**FIGURE 9:**
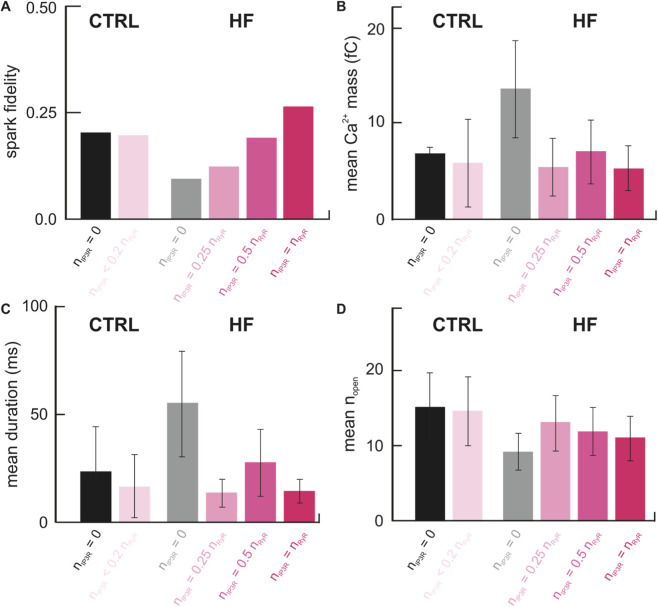
Spark characteristics from HF CRUs depend on the level of IP_3_R2 expression. **(A)** Spark fidelity is relatively the same in CTRL CRUs with and without a low level of IP_3_R2s while spark fidelity increases with increasing N_IP3R_ in HF CRUs. **(B,C)** The mean ± standard deviation total amount of Ca^2+^ released during a spark (Ca^2+^ spark mass) and mean ± standard deviation spark duration are similar between the CTRL and HF groups with IP_3_R2s. **(D)** The mean ± standard deviation total number of open channels involved (maximum n_open_) increases with increasing NIP3R in HF CRUs. Histograms showing the distributions of this data are included in Supplementary Figure S1.

We next sought to explore the implications of increasing IP_3_R2 expression on spark behavior from CRUs observed in failing ventricular myocytes. We first converted published raw images in which RyR2s were fluorescently labeled and imaged using dSTORM into RyR2 arrangements to be input into our model. For the healthy control CRUs, one IP_3_R2 was placed randomly among the clustered RyR2s to represent the low level of expression observed under normal conditions. For the failing myocyte CRUs, N_IP3R_ was set to 25%, 50%, and 100% of N_RyR_, and IP_3_R2s were again placed randomly among the RyR2s. These relative levels of IP_3_R2 expression were chosen to represent the full range of expression that may be observed based on currently available data (Lipp et al., 2000; Mackenzie et al., 2002; Harzheim et al., 2009; Wullschleger et al., 2017; Demydenko et al., 2021; Jin et al., 2022). For each set of conditions, 500 simulations were run (each simulation 200 ms) in which one RyR2 was forced open, and it was observed whether or not a Ca^2+^ spark was triggered. Resulting sparks were characterized by the total mass of Ca^2+^ released (fC), duration of the spark event (ms), and maximum number of channels opened at one time during the spark. [IP_3_] was set to 0.1 µM for all simulations (Figure 9).

Sparks from the healthy control (CTRL) CRUs occurred in 19.2% of simulations. These sparks had a mean Ca^2+^ mass of 5.85 ± 9.05 fC, a mean duration of 16.52 ± 29.33 m, and involved ∼15 ± 9 open channels on average (Figure 9). These values are similar to results in which no IP_3_R2s were present, supporting our hypothesis that a low level of IP_3_R expression under healthy conditions does not notably alter Ca^2+^ spark properties.

In HF CRUs, spark fidelity increased as N_IP3R_ was progressively increased from 0% to 100% N_RyR_ (Figure 9), consistent with our findings in Figure 8. When comparing the Ca^2+^ sparks from HF and CTRL CRUs, it was observed that in the absence of IP_3_R2s, HF CRUs had lower spark fidelity (9.2% compared to 19.8%), involved more total Ca^2+^ release during spark events (13.50 ± 10.03 fC compared to 6.81 ± 1.40 fC), and resulted in longer spark duration (55.38 ± 48.97 m compared to 24.59 ± 43.77 m) on average. These differences were not observed when IP_3_R2s were present (Figure 9). Spark fidelity in the HF group varied from 12% to 25.8% depending on N_IP3R_, whereas spark fidelity in the CTRL group with N_IP3R_ <20% of N_RyR_ fell within that range at 19.2%. Ca^2+^ spark mass ranged from 5.25 ± 4.62 fC to 7.03 ± 6.58 fC in the HF groups, compared to 5.85 ± 9.05 fC in the CTRL group (N_IP3R_ < 0.2 N_RyR_), and spark durations ranged from 13.73 ± 12.99 m to 27.78 ± 31.04 m in the HF group, compared to 16.52 ± 29.33 m in the CTRL group. Max n_open_ in the HF group varied depending on level of IP_3_R2 expression, with higher N_IP3R_ resulting in lower max n_open_. We found it somewhat unexpected that, when IP_3_R2s were incorporated, there was no evident correlation observed between N_IP3R_ and Ca^2+^ mass or spark duration, in contrast to our previous findings in CTRL and HF CRUs without IP_3_R2s, as well as recent modeling results by Chung et al. that predicted an increase in spark amplitude (similar to our Ca^2+^ mass) at higher N_IP3R_ levels (Chung et al., 2023). Rather, HF CRUs with IP_3_R2s incorporated had similar mean Ca^2+^ spark mass (Figure 9B) and duration (Figure 9C) to CTRL CRUs regardless of N_IP3R_. From these results, we further hypothesized that the increasing IP_3_R2 expression observed in HF may serve a compensatory role to make up for differences in spark behavior due to RyR2 cluster remodeling.

### 3.3 IP_3_R2 expression results in “multi-spark” events in failing myocytes

**FIGURE 10:**
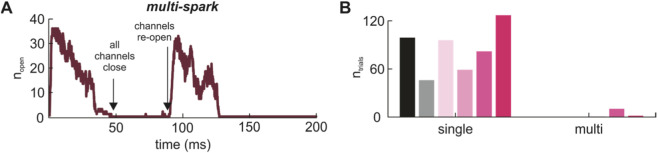
IP_3_R2 expression facilitates multi-sparks in HF CRUs. **(A)** Shown is an example of a “multi-spark” in which a spark occurs in response to one channel being forced opened, all channels close to terminate the spark, and then channels reopen later to generate another spark without any channels being forced open. **(B)** All sparks were classified as single or multi-sparks for CTRL and HF groups. Shown in the bar graph is the number of simulations out of 500 in which each type of event occurred in CTRL (N_IP3R_ = 0), HF (N_IP3R_ = 0), CTRL (N_IP3R_ = 1), HF (N_IP3R_ = 0.25 N_RyR_), HF (N_IP3R_ = 0.5 N_RyR_), and HF (N_IP3R_ = N_RyR_) groups, in order from left to right. Multi-sparks were only observed in HF CRUs with IP_3_R2s (1 out of 500 simulations for HF CRUs with N_IP3R_ = 0.25 N_RyR_, 10 out of 500 simulations for HF with N_IP3R_ = 0.5 N_RyR_, and 2 out of 500 simulations with N_IP3R_ = N_RyR_. In all simulations, [IP_3_] was 0.1 μM.

Upon further examination, simulations of HF CRUs using the *RyR-IP*_*3*_ *R model* revealed the incidence of rare “multi-spark” events (Figure 10A). In HF simulations with N_IP3R_ = 50% N_RyR_, “multi-sparks” in which two or more sparks occur within a 200-m simulation were observed in 10 out of 500 simulations. Multi-sparks were not observed in CTRL simulations (Figure 10B). Similar multi-spark events were reported by Chung et al. (2023).

## 4 Discussion

The work presented here aimed to model local Ca^2+^ release, or Ca^2+^ sparks, via both RyR2s and IP_3_R2s at SR junctions to study how subcellular remodeling of RyR2 within CRUs and increased IP_3_R2 expression in heart failure affect cardiac EC coupling. We developed an *RyR-IP*_*3*_ *R Ca* ^*2+*^ *sparks model* and ran simulations showing that (1) increased IP_3_R2 expression and [IP_3_] increase spark fidelity; (2) a low level of IP_3_R2 expression within healthy control CRUs has little effect on Ca^2+^ spark properties; and (3) increased IP_3_R2 expression in HF CRUs causes multi-spark events.

Similar to results recently presented by Chung et al. (2023), we showed that increasing the number of IP_3_R2s in proximity to RyR2s increases spark probability (Figure 8). These authors further demonstrated that these conditions lead to “Ca^2+^ leak” via stochastic opening and closing of IP_3_R2s. Ca^2+^ leak elevates the baseline dyadic [Ca^2+^], bringing RyR2s closer to their opening threshold (Chung et al., 2023). In their simulations, Chung et al. used idealized RyR2 arrangements. Our work shows that these results are also applicable to realistic RyR2 cluster geometries, which tend to be more irregular. Arrangements are based on measurements from isolated rat ventricular myocytes (Kolstad et al., 2018), which may differ from human. When similar imaging data from human ventricular myocytes become available, these will provide a valuable resource for testing the extent to which our results apply to human pathophysiology.

Despite increased spark fidelity with increasing N_IP3R_ observed in Figure 8, we were surprised to see that increased N_IP3R_ did not result in differences in spark population characteristics including total Ca^2+^ released and spark duration. Based on previous simulation results using an RyR2-only model (data not shown), we expected there to be differences in spark behavior between healthy control and HF CRUs and that these might be further exaggerated when IP_3_R2 expression is included. From this, we concluded that increasing IP_3_R2 expression in HF may play a compensatory role to make up for differences in spark behavior due to RyR2 cluster fragmentation and dispersion. Further analysis of the Ca^2+^ sparks revealed that HF CRUs with increased IP_3_R2 expression produce “multi-sparks.” In a small number of cases, the initial spark terminates and then another spark occurs shortly after with no further stimulation. Based on our simulations, there is likely a “sweet spot” around an intermediate level of IP_3_R expression that results in these multi-spark events. Some contributing factors are likely reduced SR Ca^2+^ load through leak when the number of IP_3_Rs becomes too high and activation of SERCA in response to increased intracellular [Ca^2+^]–both reducing the likelihood of additional sparks. These multi-spark events would be reflected experimentally as an increase in diastolic spark probability, and, although rare in our simulations, may have pathophysiologic significance if they facilitate the propagation of Ca^2+^ waves.

This preliminary work raises several questions which will be the focus of future work. For example, does Ca^2+^ spark behavior change in response to more robust IP_3_ pathway stimulation? How does the placement of RyR2s and IP_3_R2s relative to each other affect their crosstalk? How do other changes that occur in HF affect spark behavior? And, do changes in HF promote Ca^2+^ wave propagation?

In cardiac myocytes, the IP_3_ pathway can be stimulated by external signals such as the neurohormones endothelin-1 (ET-1) and angiotensin II, leading to downstream effects that promote hypertrophy, inflammation, fibrosis, and oxidative stress. These effects may in turn contribute to cardiac dysfunction and remodeling in conditions such as HF and myocardial infarction (Mehta and Kathy, 2007; Jankowich and Choudhary 2020). In our initial simulations with the *RyR-IP*_*3*_ *R model*, we kept a constant basal level of [IP_3_] at 0.1 µM for both HF and control conditions. However, it is important to note that different levels of ET-1 and angiotensin II signaling at progressive stages of hypertrophy and HF may lead to either smaller or greater changes in intracellular [IP_3_]. Furthermore, it might help us make better sense of our results in CTRL versus HF sparks to explore how increasing [IP_3_] affects spark behavior. We expect that increasing [IP_3_] would increase spark frequency in both CTRL and HF but that this would be much more pronounced in the HF CRUs (Jin et al., 2022).

Super-resolution microscopy techniques have been used to map the fine structure of RyR2 clusters (Jayasinghe et al., 2018; Kolstad et al., 2018; Shen et al., 2019; Asghari et al., 2020), with DNA-PAINT achieving a near single channel resolution of 40–60 nm (Jayasinghe et al., 2018). Similar studies have not yet been conducted to map IP_3_R2 locations within CRUs, and standard confocal and STED measurements have only given us a relative idea of IP_3_R2 location within cells (Harzheim et al., 2009; Jin et al., 2022). With limited immunofluorescence data, we know that IP_3_R2s are expressed at SR junctions with RyR2s. However, the exact positioning of the channels relative to one another is unknown, and this lack of detailed information required us to make assumptions in our simulations. In all simulations, we begin with a CRU containing only RyR2s and then randomly place IP_3_R2s at empty locations within them. Recent experimental results from Jin et al., however, suggest that RyR2s and IP_3_R2s may form distinct clusters that have varying degrees of overlap with one another (Jin et al., 2022). These authors concluded that this allows for IP_3_R2-mediated Ca^2+^ release to engage or “recruit” RyR2s from regions uncoupled from t-tubules due to remodeling in HF (Jin et al., 2022) and thus may be particularly relevant to the recruitment of rogue or uncoupled RyR2s which are more frequent in HF (Dries et al., 2018; Kolstad et al., 2018). Until techniques with higher spatial resolution such as DNA-PAINT (Jayasinghe et al., 2018) are applied to determine individual RyR2 and IP_3_R2 locations simultaneously, models such as ours will be required to make assumptions to explore how the relative arrangement of RyR2s and IP_3_R2s affects their crosstalk.

To create a more comprehensive model of heart failure (HF), we incorporate two key changes to excitation–contraction coupling that occur in HF: (1) decreased SR [Ca^2+^] load due to reduced SERCA activity, and (2) hyperactive or “leaky” RyR2s via beta-adrenergic and CaMKII-dependent phosphorylation and redox modification that increase RyR2 open probability. Including RyR2 phosphorylation in a model of Ca^2+^ release in HF is particularly significant, as modeling and experimental evidence suggest that phosphorylation also modulates the arrangement of RyR2s within CRUs. For instance, Asghari et al. used dSTORM super-resolution microscopy to investigate the effects of RyR2 phosphorylation on RyR2 arrangements within CRUs in rat ventricular myocytes (Asghari et al., 2020). Their findings revealed that phosphorylation rearranges RyR2s into larger, mostly-checkerboard-like clusters, and that sparks are more frequent under these conditions (Asghari et al., 2020). Furthermore, computational modeling by Hernández Mesa et al. demonstrated that the phosphorylation pattern of RyR2s further modifies Ca^2+^ spark behavior in a distribution-dependent manner, with higher spark fidelity observed when phosphorylated RyR2s are concentrated at the center of a CRU compared to when they are uniformly distributed throughout (Mesa et al., 2022). Moreover, it is important to explore how the combined effect of remodeling that disperses RyR2s and phosphorylation that promotes a more regular arrangement, as suggested by Asghari et al., may influence Ca^2+^ spark behavior in HF. These insights shed light on the complex interplay between RyR2 phosphorylation and spatial organization, and contribute to a more comprehensive understanding of the underlying mechanisms of aberrant Ca^2+^ signaling in HF.

### 4.1 Limitations

Several limitations of the present model should be acknowledged. First, our arrangements of IP_3_R2s relative to RyR2s should be considered hypothetical, since data on spatial localization of individual IP_3_R2s within dyadic CRUs are extremely limited. When placing IP3R2s, we assume that they occupy a 30 nm by 30 nm space on the SR surface, identical to the surface area occupied by RyR2s. Although RyR2s and IP_3_R2s are similar in size and share regions of sequence homology, they are distinct channel proteins with important differences in molecular weight and oligomerization properties (e.g., homo-versus heterotetramer formation) which are not explicitly represented in the current formulation. Finally, although functional coupling between neighboring RyR2s in ventricular myocytes is well-established and is thus included in our model (Rios, 2026), any coupling that may occur among IP_3_R2s or between IP_3_R2s and RyR2s has not been characterized. Future work incorporating higher resolution channel localization and interaction mechanisms may help address these limitations.

### 4.2 Future directions

Our primary interest in studying Ca^2+^ sparks is to identify the mechanisms that govern their transition into arrhythmogenic Ca^2+^ waves. Future work may benefit from extending the present framework to a spatially resolved Ca^2+^ wave model in which multiple Ca^2+^ release units interact through Ca^2+^ diffusion. Such an extension would enable mechanistic exploration of how multi-spark events and subcellular heterogeneity promote spontaneous Ca^2+^ release, linking intracellular Ca^2+^ dysregulation to delayed afterdepolarizations and arrhythmia initiation.

In summary, we present a model of local Ca^2+^ release, or Ca^2+^ sparks, that accounts for Ca^2+^ release through both RyR2s and IP_3_R2s in healthy and failing ventricular myocytes. These results highlight the importance of crosstalk between RyR2s and IP_3_R2s in modulating EC coupling in the heart, especially in the context of heart failure.

## Data Availability

The raw data supporting the conclusions of this article will be made available by the authors, without undue reservation.
