# Supplementary material for: Modeling the spatiotemporal properties of crosstalk between RyR-mediated and IP3R-mediated local Ca2+ release
Source: Front Cell Dev Biol. 2026 Apr 22;14:1736729. doi: 10.3389/fcell.2026.1736729 (PMC13144025; doi:10.3389/fcell.2026.1736729)
Supplement: Supplementary file 1 [file DataSheet1.pdf]

SUPPLEMENTAL DATA

Table S1. Model parameters

DIFFUSION COEFFICIENTS				
**VARIABLE**	**DESCRIPTION**	**VALUE**	**REFERENCE(S)**	**NOTES**
D_{myo}	Diffusion constant of Ca^{2+} within myoplasm	0.225 $\mu m^2\ ms^{-1}$	(Smith et al., 1998)	
D_{JSR}	Diffusion constant of Ca^{2+} within JSR	0.060 $\mu m^2\ ms^{-1}$	(Wu and Bers, 2006)	
D_{NSR}	Diffusion constant of Ca^{2+} within NSR	0.060 $\mu m^2\ ms^{-1}$	(Wu and Bers, 2006)	
τ_{refill}	JSR refilling time constant	8 ms	(Sobie et al., 2005)	Fixed to produce $\tau_{recovery}$ of total $[Ca^{2+}]_{JSR}$ of 90 ms
**FIXED GEOMETRIC PARAMETERS**				
w_{RyR}	RyR side width	0.030 µm	(Baddeley et al., 2009)	
h_{ds}	Dyadic space height	0.020 µm	(Rog-Zielinska et al., 2021)	
**RYR GATING PARAMETERS**				
k^-_r	Maximum RyR closing rate	1 ms^{-1}		Fixed to produce appropriate spark fidelity
$k^+_{r,max}$	Maximum RyR opening rate	1.1 ms^{-1}		Fixed to produce appropriate spark fidelity
$K_{r,max}$	Sensitivity of opening to dyadic space $[Ca^{2+}]$	19.87 µM		Fixed to produce appropriate spark fidelity
α_r	RyR luminal dependence factor	6.5×10^{-3}		Fixed to produce appropriate spark fidelity
hill	Hill coefficient	4	(Sobie et al., 2002)	
P_{RyR}	RyR permeability constant	1×10^{-12} µL ms^{-1}	(Mejía-Alvarez et al., 1999; Kettlun et al., 2003; Groff and Smith, 2008; Walker et al., 2014)	P_{RyR} of 1×10^{-12} µL ms^{-1} corresponds to single RyR channel current of 0.03 pA, which is in the range measured by experimental studies and used in similar modeling studies.
EJ	RyRs coupling	0.35	(Groff and Smith, 2008; Williams et al., 2011)	
**BUFFERING PARAMETERS**				
$[B_{CaM}]_{total}$	Total calmodulin concentration	24 µM	(Smith et al., 1998; Shannon et al., 2000)	
$[B_{SR}]_{total}$	Total SR membrane buffer concentration	47 µM	(Smith et al., 1998; Shannon et al., 2000)	
$[B_{SL}]_{total}$	Total SL membrane buffer concentration	900 µM		
$k_{CaM,on}$	Calmodulin Ca^{2+} on rate constant	100 $\mu M^{-1}\ ms^{-1}$	(Smith et al., 1998)	
$k_{SR,on}$	SR membrane buffer Ca^{2+} on rate constant	115 $\mu M^{-1}\ ms^{-1}$	(Smith et al., 1998)	
$k_{SL,on}$	SL membrane buffer Ca^{2+} on rate constant	115 $\mu M^{-1}\ ms^{-1}$	(Smith et al., 1998)	

$k_{CaM,off}$	Calmodulin Ca^{2+} off rate constant	38 ms^{-1}	(Smith et al., 1998)	
$k_{SR,off}$	SR membrane buffer Ca^{2+} off rate constant	100 ms^{-1}	(Smith et al., 1998)	
$K_{SL,off}$	SL membrane buffer Ca^{2+} off rate constant	1000 ms^{-1}	(Smith et al., 1998)	
[CSQ]	Concentration of calsequestrin in the JSR	30×10^3 µM	(Shannon et al., 2000)	Given total measured SR buffer of ~3 mM, we assumed that most CSQ resides in the JSR (~10% of the total SR volume).
K_{CSQ}	Calsequestrin Ca^{2+} dissociation constant	630 µM	(Shannon and Bers, 1997; Shannon et al., 2000)	
**BASELINE IONIC CONCENTRATIONS**				
$[Ca^{2+}]_{myo}$	Bulk Ca^{2+} concentration in myoplasm	0.1 µM	(Cheng et al., 1993)	
$[Ca^{2+}]_{NSR}$	NSR Ca^{2+} concentration	850 µM	(Bers, 2014)	Bers et al. and others estimate baseline $[Ca^{2+}]_{SR}$ within a range of 700–1,100 µM.
**SERCA PARAMETERS**				
$K_{myo,SERCA}$	Ca^{2+} dissociation constant for SERCA on myoplasmic side	910 µM	(Tran et al., 2009; Williams et al., 2011)	
$K_{SR,SERCA}$	Ca^{2+} dissociation constant for SERCA on SR side	2240 µM	(Tran et al., 2009; Williams et al., 2011)	
v_{cycle}	SERCA pump cycling rate	Varies with $[Ca^{2+}]_{NSR}$	(Tran et al., 2009; Williams et al., 2011; Walker et al., 2014)	This cycling rate assumes a myoplasm SERCA Ca^{2+} dissociation constant of 910 µM and NSR SERCA Ca^{2+} dissociation constant of 2240 µM.
[SERCA]	SERCA pump concentration in each NSR element	150 µM	(Williams et al., 2011; Walker et al., 2014)	
